# Cough Test Results during Screening for Silent Aspiration Are Affected by Risk Factors for Silent Cerebral Infarct in Older Adults with Chronic Disease

**DOI:** 10.3390/ijerph191610202

**Published:** 2022-08-17

**Authors:** Ayako Nakane, Kazuharu Nakagawa, Kohei Yamaguchi, Kanako Yoshimi, Yoshiko Hara, Haruka Tohara

**Affiliations:** Department of Dysphagia Rehabilitation, Graduate School of Medical and Dental Sciences, Tokyo Medical and Dental University, Tokyo 113-8549, Japan

**Keywords:** aspiration pneumonia, cough test, older adults, screening test, silent cerebral infarct

## Abstract

The cough reflex does not change with age. However, older adults with chronic diseases often have a reduced cough reflex. The effects of several risk factors on reduced cough sensitivity in older adults remain unclear. This study aims to clarify the risk factors for reduced cough sensitivity in older adults with chronic diseases. This cross-sectional study included participants aged <65 years (young group; *n* = 21), those aged ≥65 years (older adults with chronic disease group; *n* = 18), and those with dysphagia (dysphagia group; *n* = 16). A cough test was performed on all participants using an ultrasonic nebulizer with a mist of 1% *w*/*v* citric acid physiologic saline. Cough response was observed in the young (21/21), older adult (9/18), and dysphagia (13/16) groups. The difference between the young and older adult groups was significant (*p* < 0.01). The older adult and dysphagia groups had decreased cough sensitivity compared to the younger group. Cough sensitivity was affected by risk factors for silent cerebral infarct and age. Our findings show that cough test results might be affected by risk factors for silent cerebral infarction in older adults with chronic diseases.

## 1. Introduction

Silent aspiration is a major cause of aspiration pneumonia in older adults [1]. Although videofluoroscopic examination of swallowing (VF) and videoendoscopic examination of swallowing (VE) are required to diagnose silent aspiration related to oral intake, we devised a cough test (CT) that is simpler than VF or VE [2]. Furthermore, this test is noninvasive, unlike VF and VE. This method for detecting silent aspiration uses a stimulus that induces the cough reflex, which is a protective response of the respiratory tract, and has good sensitivity and specificity to screen for silent aspiration [3,4].

The cough response threshold has been reported to increase in patients with aspiration [5]. Further, some reports found that the cough reflex is delayed in patients with dysphagia [6]. The cough reflex does not normally change with age [7]. However, we often see a decreased cough reflex in older adults with chronic disease during CT. Moreover, it has been reported that older adults often have a reduced cough reflex [8]. Older adults and patients with dysphagia may have various diseases, among which the presence of a silent cerebral infarct (SCI) in the basal ganglia region has been reported to reduce the cough reflex [9]. The risk factors for SCI include renal dysfunction, hypertension, diabetes, and heart disease [10,11], which are often evident in older adults [12]. SCI is also reported to be a cause of aspiration pneumonia [13]. Therefore, CT results in older adults with chronic disease and patients with dysphagia might be affected by SCI. In this study, we performed CT using citric acid, which is widely used in screening tests for silent aspiration, in young individuals, elderly individuals with chronic disease, and patients with dysphagia, to investigate whether the results were affected by the risk factors of SCI.

## 2. Materials and Methods

### 2.1. Participants

The participants were older adults (≥65 years old) with and without dysphagia who were receiving outpatient care at Tokyo Medical and Dental University Dental Hospital, as well as younger participants (<65 years old) affiliated with Tokyo Medical and Dental University. Individuals who were independent in their daily lives, had no problems communicating, and agreed to participate in the study were selected.

The participants with no symptoms of dysphagia were divided into two groups: young group (*n* = 21, 5 men and 16 women, 32.0 ± 8.7 years of age, with hypertension in 1 [5%] participant) and older adult group (*n* = 18, 7 men and 11 women, 78.1 ± 6.4 years of age, with chronic diseases, including heart disease in 6 [33%], diabetes in 4 [22%], ischemic stroke in 3 [17%], dementia in 3 [17%], and hypertension in 2 [11%] participants). The patients with dysphagia (*n* = 16, 10 men and 6 women, 75.6 ± 10.7 years of age) had a swallowing disorder caused by several diseases (oral cancer in 9 [56%], ischemic stroke in 2 [13%], hypertension in 1 [6%], brain tumor in 1 [6%], stomach cancer in 1 [6%], heart disease in 1 [6%], and Parkinson’s disease in 1 [6%] participant). Patients who were undergoing chemotherapy or radiation therapy, had a tracheotomy, had asthma, or were taking antitussive agents were excluded. In addition, oral cancer patients were included if they had undergone surgery at least 3 months earlier and records showed that the superior laryngeal nerve had not been resected. The medical history was extracted from the medical records.

As dysphagia can be classified into stages (anticipatory, masticatory, oral, pharyngeal, and esophageal) with different pathophysiologies, patients were classified by dysphagia stage. The dysphagia group included 8 (50%) patients with oral dysphagia and 8 (50%) with pharyngeal dysphagia. The Dysphagia Severity Scale (DSS) [14] scores of participants in the dysphagia group were 2 in 3, 3 in 4, 4 in 1, 5 in 5, and 6 in 3 participants. The participants with dysphagia were selected from those diagnosed with VF or VE in the dysphagia rehabilitation department. This study was approved by the Ethical Committee of Tokyo Medical and Dental University (No. D2018-008) and was performed after we explained the purpose and procedures of the study to the participants and obtained their written consent. This study was conducted according to the principles of the Declaration of Helsinki.

### 2.2. Methods

This cross-sectional study was performed at the Tokyo Medical and Dental University and dental hospital in October 2018. A CT was performed for all participants using a mist of 1% *w*/*v* citric acid and physiologic saline for 1 min with an ultrasonic nebulizer (NE-U22; Omuron, Kyoto, Japan). Participants were directed to breathe through the mouth. An observer recorded the number of times the participants coughed during the 1 min of nebulization. The time from the start of inhalation to the first cough was measured as cough latency in 60 s. If the participant did not cough during the test, the test was concluded, and the participant was confirmed as having an absence of cough response. During statistical processing, the cough latency was considered as 60 s, and the frequency of cough was recorded as 0 n/min.

We obtained the participants’ medical histories and information on their difficulty in swallowing from interviews or medical records. We also considered the presence of renal dysfunction, hypertension, diabetes, and heart disease as significant risk factors for SCI [10,11]. We conducted a power analysis using the G*Power 3.1 software (Kiel University, Kiel, Germany). The required sample size was calculated with a detection power of 0.8, an alpha of 0.05, and an effect size f of 0.5. The analysis of variance test required at least 42 participants. A total of 55 participants were enrolled. Using a post hoc test, the detection power was calculated as 0.99 depending on the latency time of each group.

### 2.3. Data Analysis

Quantitative variables are expressed as means ± standard deviations. Categorical data are expressed as frequencies (percentages). Differences between the groups were adjusted for multiplicity using the Kruskal-Wallis test and Fisher’s exact test, followed by the Bonferroni test. A multiple regression analysis (forced entry method) was used to examine the effect of independent variables on cough frequency and latency. The selection of priority variables was based on the previous literature. Oral dysphagia was excluded because it was not directly related to aspiration [15]. Medical history was incalculable and was, therefore, not included in the multiple regression analysis. Logistic regression analysis (forced entry method) was performed to determine the effect of independent variables on the presence or absence of cough. In the logistic regression analysis, although the number of cases was insufficient, we included three independent variables. All statistical analyses were performed using SPSS version 24 (IBM Japan, Tokyo, Japan). Statistical significance was set at *p* < 0.05.

## 3. Results

### 3.1. General Characteristics of Patients

Table 1 shows the participants’ background. The difference in age between the young and older adult groups as well as that between the young and dysphagia groups was statistically significant (*p* < 0.01); however, there was no significant difference in age between the older adult and dysphagia groups.

There was a difference in the average number of medications being taken by each group, with the older adult group taking the highest number of medications at 5.3 (*p* < 0.01). There were three and two participants with a history of stroke in the older adult and dysphagia groups, respectively; however, there was no statistically significant difference between the groups. There was a statistically significant difference in the medical history between the young and older adult groups as well as between the young and dysphagia groups (*p* < 0.01). The older adult group had the highest number of participants with the presence of risk factors for SCI in their medical history, and the difference between the older adult and young groups as well as between the older adult and dysphagia groups was statistically significant (*p* < 0.01).

### 3.2. CT Results

The results of the CT are shown in Table 2. All participants in the young group had a cough response (21/21 participants (100%) vs. 9/18 participants (50%) in the older adult group; *p* < 0.01). There was no difference in the number of participants with cough reflex between the dysphagia (13/16 participants (81%)) and young groups.

### 3.3. Cough Latency during CT

The results of cough latency during CT are shown in Table 2. The young group had a shorter time (2.4 ± 0.9 s) until the appearance of the first cough than the older adult (42.9 ± 24.0 s) and dysphagia groups (30.0 ± 22.6 s) (*p* < 0.01); there was no significant difference between the older adult and dysphagia groups.

### 3.4. Cough Frequency during CT

The results of cough frequency during the CT are shown in Table 2. The young group (5.0 ± 0 n/min) had more coughs per minute than the older adult (1.8 ± 2.3 n/min) and dysphagia groups (2.7 ± 2.1 n/min) (*p* < 0.01); there was no significant difference between the older adult and dysphagia groups.

### 3.5. Factors Affecting CT

When cough latency on CT was adjusted for age, number of medications, stroke history, dysphagia of pharyngeal stage, and risk factors for SCI based on the results of multiple regression analysis, the risk factors for SCI (β = 0.49, *p* = 0.03) and age (β = 0.38, *p* = 0.02) had a significant effect and were independently related factors. Similarly, when cough frequency was adjusted for age, number of medications, stroke history, dysphagia of pharyngeal stage, and risk factors for SCI based on the results of multiple regression analysis, the risk factors for SCI (β = −0.37, *p* = 0.03) and age (β = −0.40, *p* = 0.02) had a significant effect and were independently related factors (Table 3).

The results of logistic regression analysis on the presence/absence of cough reflex revealed the risk factors for SCI (Table 4).

## 4. Discussion

### 4.1. Cough Sensitivity Decreases in Older Adult Patients and Patients with Dysphagia

Previous reports have shown that the cough reflex in healthy individuals does not change with age [7]. However, a study found that the sensitivity of the cough reflex was significantly reduced in older adults, even among those with adequate respiratory function, in a comparison of 83-year-old participants with 27-year-old participants [8]. Another report comparing a healthy young group with an older adult group found that cough latency in the older adult group was longer than that in the young group, at 14.91 s [6].

The results of this study show that the older adult group had a longer cough latency than the young group by 3.96 s. A previous report on healthy older adult participants found that there was no age-related change in cough sensitivity [7]. The older adult group with a significantly reduced sensitivity of cough reflex included participants with other health conditions. In the report [6] where the cough latency of the older adult group was 14.91 s, the average age of the older adult group was 63.66 years, which is 15 years younger than the average age of our older adult group. The participants selected in our older adult group were not healthy participants, but those with various diseases. The average age of the older adult group was 78.1 years, and the average number of medications being taken was 5. It has been reported that 86% of the older people aged 75 years and above have a chronic disease and take one or more medications, while 64% have two or more chronic diseases for which they take medications (Tokyo Metropolitan Association of Medical Care services for older senior citizens, 2015). This indicates that the older adult group in our study is representative of the general population. We found that recruiting a representative older adult cohort was necessary to examine factors other than age that affect cough sensitivity.

Reports have shown that the threshold of cough response is high in patients with aspiration pneumonia [5]. The dysphagia group had a longer cough latency and lower cough frequency than the young group, but there was no statistically significant difference between the dysphagia group and the older adult group. There was no statistically significant difference in age between the dysphagia and older adult groups; all participants had medical histories, and the proportion of participants with a history of stroke was also similar. The dysphagia group took an average of two medications; thus, there was a difference in the number of medications compared to the older adult group. However, the number of medications was not an independent factor affecting cough sensitivity in multivariate analysis. A previous study reported the cough latency of their dysphagia group with a mean age of 72.95 years as 29.61 s; this was similar to the mean age of the participants and the results of cough latency in our study [6]. Moreover, pharyngeal disorder was not listed as a factor affecting cough sensitivity in that study.

This study revealed that older adults with chronic diseases and patients with dysphagia had a decreased cough sensitivity compared to that of the young group, and age and risk factors for SCI affected cough sensitivity to a greater extent than the number of medications and pharyngeal disorders. Considering that the risk factors for SCI increase with age [12] and that cough reflex is maintained in healthy older adults [7], it is possible that SCI in older adults and patients with dysphagia might reduce cough sensitivity. Therefore, a decreased cough reflex is more likely to be observed in older adults with chronic disease undergoing a CT.

### 4.2. Risk Factors for SCI and Absence of Cough Reflex

In this study, 50% of the participants in the older adult group and 19% in the dysphagia group had no cough response. Reports have found that SCI occurs in 42–62.1% of older Japanese adults [16,17]. Many of these infarcts are basal ganglia lesions, and it has been reported that the swallowing reflex is affected and pneumonia is more likely to occur in the presence of these lesions [18]. There are several reports on the absence of a cough response in older adults [4], but the mechanism is not fully understood. However, the proportion of SCI in the Japanese older adult population and the proportion of participants with the absence of a cough response in our older adult group were similar. Furthermore, in this study, the percentage of participants with no cough response was higher in the older adult group than in the dysphagia group, and while there was no statistically significant difference in medical history in both groups, the risk factors for SCI were higher in the older adult group. We did not compare the brain CT findings in this study; thus, the number of patients with risk factors for SCI who had actual SCI findings are unknown. However, the results of the multiple regression analysis in this study listed that hypertension, diabetes, chronic renal dysfunction [10], atrial fibrillation [11], and SCI are also known to increase with age. The logistic regression analysis also showed that the adjusted odds ratio for the risk of SCI was significantly high, depending on the presence or absence of a cough response. On comparing the older adult and dysphagia groups, there was a difference in the number of medications and the risk factors for SCI. Multiple regression analysis showed no effect of the number of medications on cough sensitivity. The only difference between the two groups was the ratio of no cough response. This suggests that risk factors for SCI affect the cough reflex. Therefore, the CT could be used for screening for SCI.

Based on the above findings, it can be assumed that cough sensitivity tends to decrease with age in older adults with chronic diseases. Cough reflex might be involved with multiple age-related factors, and of these factors, the presence of risk factors for SCI may have a strong influence. We assume that older adult patients with multiple chronic illnesses would have SCIs, and that these may contribute to the lack of cough. Cough reflex needs to be considered separately for the healthy older adults and older adults with diseases. In research studies, it is important to recruit healthy older adults to understand the effect of aging alone. However, clinically, a CT is typically performed on unhealthy older adults who are at risk for SCI. It is, therefore, important to understand the factors affecting cough reflex to avoid aspiration pneumonia. We believe that identifying the absence of cough may increase awareness of the risk of aspiration pneumonia in our rapidly aging population and may make a notable contribution to maintaining quality of life in older adults while also saving medical costs. Therefore, in future studies, we would like to elucidate the relationship between brain imaging findings and CT in older adults.

### 4.3. Limitations

There are several limitations in this study. Since the participants were recruited from a Dental Hospital, there were many patients with dysphagia caused by oral cancer surgery. Patients were outpatients several months post-surgery, which may have affected the study results. In this study, 50% of the participants in the older adult group and 19% in the dysphagia group had no cough response. This affected the statistical processing of cough latency in the participants with no cough. Therefore, the cough latency for a person with no cough response was set as 60 s. This might have lengthened the cough latency time. Moreover, this study only counted the number of oral medications and did not examine their type or effects; hence, the effects of medications on the results cannot be ruled out. Finally, there were imbalances based on sex in all participant groups.

## 5. Conclusions

This study implemented a CT using citric acid, which is used as a screening test for silent aspiration. Due to chronic health factors associated with aging, cough latency and cough frequency are assumed to decrease in older adults. This study demonstrated that age and risk factors for SCI affect the results of CT in older adults with chronic diseases.

## Figures and Tables

**Table 1:** General characteristics of all participants (*n* = 55).

	Young	Older Adult	With Dysphagia	*p*
	*n* = 21	*n* = 18	*n* = 16	
Age (years)	32.0 ± 8.7	78.1 ± 6.4	75.6 ± 10.7	**
Female, *n* (%)	16 (76.2)	11 (61.1)	6 (37.5)	0.058
Medicine, mean ± SD	0.4 ± 1.6	5.3 ± 2.2	2.3 ± 2.6	**
Stroke history, *n* (%)	0 (0)	3 (16.7)	2 (12.5)	0.167
Medical history, *n* (%)	1 (4.8)	18 (100)	16 (100)	**
Risk factor for SCI, *n* (%)	1 (4.8)	17 (94.4)	5 (31.3)	**

SCI, Silent cerebral infarct; SD, Standard deviation; ** *p* < 0.01.

**Table 2:** Outcomes of all groups of the cough test.

	Young	Older Adult	With Dysphagia	*p*
	*n* = 21	*n* = 18	*n* = 16	Young vs. Older Adult Older Adult vs. with Dysphagia with Dysphagia vs. Young
Presence of cough, (%)	21 (100)	9 (50.0)	13 (81.3)	0.000 0.239 0.216
Cough latency, (min)	2.4 ± 0.9	42.9 ± 24.0	30.0 ± 22.6	0.000 0.628 0.000
Cough frequency, (n/min)	5.0 ± 0	1.8 ± 2.3	2.7 ± 2.1	0.000 0.681 0.012

Cough latency: Time from the start of the inhalation to the first cough; Cough frequency: Number of times the participants coughed during the 1 min of nebulization; *p*-value: adjusted for multiplicity by Bonferroni correction.

**Table 3:** Results of multiple regression analysis.

**Results of Multiple Regression Analysis for Cough Latency (*n* = 58)**						
	**B**	**95% CI**		***β***	***p***	**VIF**
		**LL**	**UL**			
Age	0.410	0.072	0.748	0.384	0.018	2.496
Medicine	−0.415	−2.823	1.993	−0.048	0.731	1.935
Stroke history	−16.928	−36.081	2.226	−0.195	0.082	1.211
Dysphagia of pharyngeal stage	0.712	−15.507	16.932	0.010	0.930	1.306
Risk factor for SCI	25.332	9.032	41.633	0.493	0.03	2.505
Adjust R^2^ = 0.51, analysis of variance *p* < 0.001						
**Results of Multiple Regression Analysis for Cough Frequency (*n* = 58)**						
	**B**	**95% CI**		***β***	***p***	**VIF**
		**LL**	**UL**			
Age	−0.037	−0.069	−0.006	−0.401	0.022	2.496
Medicine	0.026	−0.202	0.253	0.034	0.820	1.935
Stroke history	0.490	−1.320	2.300	0.064	0.589	1.211
Dysphagia of pharyngeal stage	0.359	−1.173	1.892	0.058	0.640	1.306
Risk factor for SCI	−1.685	−3.225	−0.144	−0.374	0.033	2.505
Adjust R^2^ = 0.43, analysis of variance *p* < 0.001						

CI, Confidence interval; LL, Lower limit; UL, Upper limit; B, Partial regression coefficient; β, Standardized partial regression coefficient; VIF, Variance inflation factor; SCI, Silent cerebral infarct.

**Table 4:** Results of the logistic regression analysis for the presence or absence of cough (*n* = 58).

Variable	OR	95% CI		*p*
		LL	UL	
Risk factor for SCI	18.864	1.530	232.643	0.022
Age	1.037	0.955	1.127	0.389
Stroke history	0.492	0.063	3.834	0.498

Percentage of correct classifications: 81.8%; CI, Confidence interval; LL, Lower limit; UL, Upper limit; OR, Odds ratio; SCI, Silent cerebral infarct.

## Data Availability

Not applicable.
